# Gleanings from Private Practice

**Published:** 1880-09-20

**Authors:** C. C. Vanderbeck

**Affiliations:** N. J.


### GLEANINGS FROM PRIVATE PRACTICE.

BY C. C. VANDERBECK, M.D., PH.D., N. J.

What a peculiar attraction clinical reports or lectures have for the mass of medical readers! It were folly to debate the value of such reading. All know the peculiar opportunities of hospital study and work; and many of the best modes of treatment of private practice of to-day, both surgical and medical, as well as numerous instruments and appliances, had their origin in the wards of hospitals. Yet, is it not true that all over our broad land many a medical expert lives and plies his vocation in small towns and rural districts? Is it not true that many a peculiar and interesting case occurs in the countryman's practice?—one that would prove of interest even to city physicians as well as to some fellow rural doctors? Who has talked with different doctors of the country but has found out that they often possess novel ideas, and good ones too, of treatment, or of some surgical appliance? So, Mr. Editor, while you give space for the "clinics," will you for a time let me seek to group on paper such useful ideas, hints and operations as I may gather from physicians removed from clinics and hospitals.

During a recent conversation with Dr. Junkin, of Easton, Pa., some useful ideas were given as regard women's diseases. In using nitrate of silver for the inner membrane of the womb, in lieu of a porte caustic, he uses a platinum wire of sufficient length, coating the end thoroughly with the silver, and in this manner:

Grasping a piece of silver nitrate with a pair of tweezers, hold it near a flame from a spirit lamp, while the opposite hand holds the end of the wire in the flame until hot enough to pierce for some distance the silver. Allowed to cool the nitrate will be found firmly fixed on the wire.

In order to make this point of proper size and shape, pass it through the flame quickly, which softens the medicine, and allows it to run as the wire may be turned or held. This part of the procedure is the most difficult, for unless care is used, the entire piece of silver will fall off. After a few trials, however, one will become expert in making these points.

In the application of this to the inside of the uterus the wire must be properly curved, and quickly passed through the neck into the body of the womb. Any delay in the neck causes immediate contraction and foils or makes difficult the passage of the instrument into the body.

The way the Doctor came to describe to me this instrument is this: I was asking advice in a case of severe and protracted menorrhagia. Examination proved the trouble to be in the uterine mucous membrane. In such cases he has had excellent results from these points, used once a week. I tried it, and so far with gratifying results, having arrested the bleeding for three weeks—the longest period for a year or more.

Dr. Junkin thinks that menstruating females are not cleanly enough. After each period tepid water should be injected high into the vagina washing out all debris. He thinks that the irritation of retained clots and mucus is one of the causes of uterine disease. Does this really not look reasonable? How many do it? How many physicians advise it? In maidens, of course, it is a little inconvenient to use a syringe, but the efforts put forth will certainly bring back good interest in health and hardiness. We are living in a hygienic age. More attention is given every year to the care of health and prevention of disease. Will not our teachers of hygiene insist upon cleanliness of the sexual organs? Yet, it will be as hard to get it done, I imagine, as it is to get people generally to use the morning cold sponge bath. Can anything be more grateful than the glow and vigor obtained from this morning application of water? It is quite possible, if we heard more of water for the uterus, we would have less to do with platinum, zinc, and nitrate of silver.

Dr. Junkin recommends young physicians to use the bivalve speculum. He says the common glass ones often discourage the inexperienced. With them it is more difficult to find the os, and to explore the uterine cavity, especially if there be any malposition.

There is a class of women in the country who get through their confinement cases without medical aid, depending upon the old women, especially those who have had "many of them," of the vicinity. Just such cases as fall to the lot of the student to learn from in the cities, or are attended by the district physicians. All is well that ends well, but if it don't end well what then? In other words, if by the lack of medical aid and attention a life is sacrificed, it may be the mother, or it may be the child, where does the responsibility rest? Should not every township have its recognized "poor" doctor, who would attend such cases for a small cash fee, to be paid in all possible cases by the family. It should be obligatory on all poor families to secure the services of a physician; if very poor, to obtain the required services at poor rates; if in very destitute circumstances, to obtain town aid. That is, no confinement case should be allowed to occur without the services of a medical man. Less of these "no doctor" cases would happen, I feel sure, if the patient knew that services could be secured at a small price without the stigma of becoming a pauper.

In our township of Upper Freehold, no regular poor doctor is appointed by the committee. The pauper can select a physician, and the overseer then arranges for attendance. Or the family calls for its physician, and he, for protection, asks the town aid for them. Charity attendance needs a little regulation in the country as well as in the city. It seems to me, the remedy is the appointment of a town physician by the committee, and after it is clearly shown that a family is unable to pay regular fees, that they may secure the advantages of the poor rates, which should be very much reduced from regular charges.

A late case in my practice will serve to illustrate the evil of no attendance in confinement.

About midnight, not long since, I was called to see an Irish woman in labor. While riding to the house the boy who came after me remarked that the women thought the child was dead, but he could tell me nothing more of the case. I found on arrival that the baby, a large, well formed female child, was born and dead. The old women said it was born after the boy started to town for me, and was dead when it came into the world. I came to the conclusion that the birth occurred before the boy left at all for me. The women had mismanaged in some way, and finding a dead child in their hands, send for a doctor, so as to secure at any rate a burial certificate.

Will some of your readers give me the probable cause of the prominence of one eye just previous to an epileptic attack, in a case of mine? I do not mean a few moments before, but it often serves as a warning one or two days before the attack. It is true it is not always followed by a "grand mal," but it is a premonition of the epileptical "bad days."

It is well known that epileptics enjoy periods of variable length of comparative freedom from unpleasant symptoms, followed by days of mental and physical distress—symptoms of reflected nervous irritation in almost all the organs of the body, diarrhœa, nausea and vomiting, backache, headache, dizziness, "petit mal," and mental feebleness. Even as a precursor of these evil days have I noticed, in the case referred to, the prominent left eye, giving a peculiar and alarming expression to the face.

Tanner, in his most excellent "Manual of Clinical Medicine and Physical Diagnosis," under the caption of "Signs Presented by the Eye," says, "The eye may be increased in size, from hyperæmia of its tissues, such as takes place in impending suffocation, or in congestion of the brain, heart, or lungs; it also becomes more prominent, and therefore apparently increased in size, in convulsions, apoplexy, epilepsy and delirium tremens."

Now, what I want to know is, what is meant by the eye being "more prominent, and therefore apparently increased in size?" Is there turgidity of the surrounding tissues in the condition causing this prominence of the eye? Will this symptom guide me to a suitable plan of treatment to relieve the sufferings of the "bad days?"

A late number of the American Agriculturist considers the subject of "Fatal Accidents from Mowing Machines," especially those resulting from drivers being thrown off from their seats in front of them. Country physicians can substantiate these statements, not only as regards the frequency of accidents to the driver, but to children who are playing in the field and running behind the machine. Two sad accidents occurred to girls in our vicinity, last summer, in this way, the foot being severed from the leg in each case.

As remarked before, this is an age of hygiene; and noble efforts are now put forth to prevent disease. What shall we call efforts to prevent accidents? It is a subject worthy of more attention than has been given it. Should not physicians carefully examine all instruments and mechanisms dangerous to life, and see whether their danger could be diminished without any special curtailment of use and power? The Agriculturist says that a lady of Burlington, N. J., invented an arrangement for throwing the knives of a mowing machine out of gear the instant the driver's weight was taken from the seat. It knows of no instance of it being put to use. Could not the inspecting and reporting of the different machines, their respective dangers, and how they could be thus improved, be the duty of boards of health ?

It has been a matter of surprise to me that country physicians do not make more of the various operations on the teeth. As a rule, they are not adequately supplied with extracting instruments, and by reason of this, so often fail to perform a good job at extracting, that they become timid, and hesitate to undertake the work. A few carefully selected instruments, including a good root, right and left molar, and an incisor forcep, will make one master of the majority of cases. As some one in a recent issue of the Reporter suggested, we should not be timid and cowardly about the various minor operations. Especially in districts without a dentist, should the doctor be prepared to relieve suffering by lifting out an offending tooth. But more, why, in such regions, could not the physician save teeth that the ignorant patient asks to have removed, by filling them with some of the usual preparations ? I am not asking a doctor to turn dentist; but the doctor can pick up many an extra dollar by being not too particular in his work, in fact, by performing his whole duty, saving tissue, relieving suffering and deformity, bestowing comfort, aiding nature.

Let us illustrate : A bright damsel came to my office to have four badly decayed teeth removed, which done, she asked if I could not clean her teeth. That is, she wanted the ugly collection of tartar on the upper front teeth removed. I went to work with the end of a match and some very fine powdered pumice stone, and using care not to injure the enamel, in less than an hour the teeth were looking clean and polished: and she was greatly pleased with the change, and paid me an extra dollar or two for my services. In small cavities, where gold is not required, and no "building up" necessary, why cannot a doctor easily learn to put a simple amalgam filling ? Often being asked by my patients after extracting, what is good for the teeth as a dentifrice, I show them a tooth powder, put up in what is called a magnesia bottle, and usually sell them a bottle, and thus further add to my income.

Gentlemen, I am not ashamed to write of little things, if I can suggest to the struggling, plodding, hard-worked (or under-worked), little-paid doctor how to add to his pocket-book some dollars.

God bless the country doctor! Yea, often is it God pity the country doctor! No thousand dollars fee ever drops into his coffers. Even down to the sale of a bottle of tooth powder must he descend for that which is to keep his family in even a degree of comfort. Wake up then, brother, glean all you can. It is all legitimately your due.

The dentifrice I referred to above is :

℞ French prepared chalk..................................
Powd. borax..................................aa ℥ viij
Powd. orris root.................................. ℥ ij
Powd. cinchona bark.................................. ʒ iv
Powd. castile soap.................................. ʒ iij
Oil wintergreen sufficient to impart an agreeable perfume.

Before closing, let me give a few formulæ I have found useful.

Great account is now made of "Rock and Rye," and advertisements are constantly seen in the papers. I make my own, thus:

℞ Rock candy........................................ 1¾ lb  

Boiling water........................................ 1 pint  

Best rye whisky........................................ 2 quarts

Stir, and after the candy is dissolved, or saturation is reached, strain.

A good liniment for some purposes, especially where iodine is useful, as in tumors, etc., is this:

℞ Iodine........................................ gr. x  

Alcohol........................................ ℥ iv  

Water of ammonia........................................ ℥ j  

Camphor........................................ ʒ ij  

Tinct. red pepper........................................ ℥ iv  

Ol. lavand........................................  

Ol. rosemary........................................aa ʒ j M

To make a successful worm liquid, add santonine to fl. ext. of spigelia and senna.

I have found sol. acetate of ammonia, in tablespoonful doses every hour until the pain is relieved, an excellent remedy for dysmenorrhœa. Sometimes the stomach is so irritable that I cannot carry out this plan to full effect, but where it is retained it has given great satisfaction.

I am using very much of the deodorized tincture of opium, and find it superior by far to any other preparation of opium. Scarcely ever does it produce nausea and headache. It is more reliable in strength than laudanum as usually kept by druggists. Many druggists make their laudanum, one ounce of opium to the pint of dilute spirits. The officinal directions are, two ounces and a half of opium to two pints of dilute alcohol.

It seems to me that we do not appreciate the value of nux vomica as we should. I have carefully examined a file of 7000 prescriptions in a country drug store, and not one called for nux vomica. I can testify to the valuable tonic properties of the following:

℞ Tinct. cincho., co........................................ ℥ iv  
Tinct. nucis vomicæ........................................ ʒ j M

Dose, one drachm three times a day.

I know not whether it is generally known that spirits of nitre is a good solvent of salicylic acid:

℞ Salicylic acid........................................ ʒ ij  

Spirits of nitre........................................ ℥ j M

Dose, one to two drachms.

I think it is often forgotten that corrosive sublimate is moderately soluble in cold water, and that dilute alcohol, or even a weaker spirit still, will dissolve it. Thus in bed-bug poison (trusting the reader may not have occasion for practical use of it) a cheap mixture, and an efficient one is:

℞ Corrosive sublimate........................................ 1 ounce  
Chloride of ammonia........................................ ½ ounce  
Dilute alcohol........................................ 1 quart

Corrosive sublimate forms, with chloride of ammonium and chloride of sodium, compounds which are more soluble than the uncombined mercurial salt. It is on this account that aqueous solutions of sal ammoniæ, or of common salt, dissolve much more corrosive sublimate than simple water. The above formula could be changed to one quart of water, and yet be found to accomplish the solution of the poison.

A good mucilage for office use is made thus :

| | | |
|---|---|---|
| ℞ | Gum arabic........................................ | 1 ounce |
| | Glycerine........................................ | 2 drachms |
| | Boiling water........................................ | 3 ounces |

I suppose every country physician keeps a number of recipes written in a little blank book for reference. In this same book, anything new he hears or reads of is put down. The objection to an ordinary book is the difficulty in finding just the formula wanting when in a hurry. I find a small-sized book-keeping ledger very convenient, and make an entry in the alphabethical pages, of every formula or item copied into the book. For instance, in "T" is Tooth Powders, page 40, and on turning to this page, the formula given above is found.

Try this plan, and find what a convenience it is, and what a pleasure it gives to have one's knowledge thus classified. It is somewhat after the style of Todd's advice to make an "Index Rerum" of all things of importance in one's reading or experience. In this manner may the work of a lifetime be saved, controlled and reviewed.—*Med. and Surg. Reporter.*

